# Scarlatina in the Bahamas

**Published:** 1846-05

**Authors:** Frederick Duncome

**Affiliations:** Nassau, Bahamas


*Scarlatina in the Bahamas. Supposed Latency of the Disease for Eleven Weeks.* By FREDERICK DUNCOME, Esq , M. D., Nassau, Bahamas.—The following circumstances will probably be deemed of sufficient importance and interest to merit a place in the Lancet, as illustrating the extent to which the latent period of scarlatina may be protracted. In this colony, the disease in question is not known to have occurred for the last forty years.

During the past summer, this malady had prevailed in some of the American cities, to an alarming and fatal extent. Amongst the sufferers was a family belonging to this place, then on a visit to Philadelphia. One young man was cut off by it, and of the four daughters, three took the disease. The youngest, aged about six years, had been secluded from the others for six weeks in a separate house, and escaped the fever. Every precaution was taken to prevent fomites. Three weeks were spent in America after the reunion of the family, and after a fortnight's passage, they arrived at this place on October 13th. Upon the 20th of December the youngest girl was attacked with "scarlatina anginosa." This case was seen by Dr. Chipman and myself, and was succeeded by some of the usual sequelæ, as otorrhœa, wry neck, œdema, and albuminous urine, the latter being marked by inflammatory symptoms.

In this history, fourteen weeks elapsed between the time our little patient was restored to the company of her convalescent sisters, and the subsequent date of the fever breaking out in her. At any rate, we have an interval of eleven weeks between the period of embarkation from America, and the breaking out of the disease in this place; so that, for at least eleven weeks, and probably longer, the infection must have remained latent in her system. Upon minute inquiry, there does not appear any reason to suppose that the malady was introduced into this place by fomites. This case did well, and the fever has not as yet spread.—*Ibid.*

